# Some Remarks on Excision of the Knee-Joint

**Published:** 1910-01-29

**Authors:** 


### SOME REMARKS ON EXCISION OF THE KNEE-JOINT.

The knee-joint is rarely excised for any other condition than advanced tuberculosis, and even in this case the result can only be looked upon as the best possible under the circumstances. The most ardent enthusiast could hardly regard it as ideal. There are those who argue that the joint should be excised as soon as it is certain that the ends of the bones are attacked, on the ground that the best possible result that can then be obtained by expectant treatment is a stiff knee, and that the patient can never regard himself as free from the danger of a recurrence unless the diseased tissue has been removed, since a tuberculous focus can only be regarded as quiescent and never as definitely cured. One must admit that the only possible treatment of a knee-joint completely disorganised by tuberculosis, as it becomes in the late stages, is excision. If it is granted for the sake of argument that the bones unite firmly and without deformity, the patient is left with a rigid and shortened limb, which is a serious handicap. But it is not always that even such a good result as this is obtained, as will be shown later.

The obvious moral is that every possible effort should be made to abort the disease in its earlier stages by recognising the condition at its commencement, by starting appropriate treatment at that time and by persevering in it. The treatment should be directed mainly to increasing the resistance of the patient to the tubercle bacillus. But it must be admitted that cases are met with in which the patient appears to have little or no resistance, and the local condition steadily progresses in spite of every care. This is often found in individuals of the very fair type, who are more or less devoid of pigment. In these excision must nearly always be resorted to sooner or later.

The operation is one whose actual technique presents little difficulty: the ultimate result depends largely on the after treatment. But there are certain points in which the procedures of different surgeons vary slightly. It is generally acknowledged that the best incision is a curved one starting from the most prominent part of one or other condyle and proceeding in a curve downwards across the centre of the ligamentum patellæ up to a similar point on the opposite side. The first question to be decided is whether the patella shall be taken away or not. In the case of a tuberculous joint this must of course always be done if the patella is involved in the disease. But even if this is not the case there is little point in leaving it, since it can no longer serve its function, for the patella is a sesamoid bone developed in the tendon of insertion of the quadriceps extensor, and after excision of the joint this muscle is no longer called into action. In any case, however, it is well to open the joint above the upper level of the patella, and to leave this temporarily attached to the tibia by its ligament, since it forms a useful means of keeping the tibia steady while the upper end is being sawn off.

The opening in the joint must be enlarged by a free division of the lateral structures, including the ligaments, and if the limb is now fully flexed the ends of the bones will be adequately exposed. The articular ends should now be sawn off and all the diseased tissue removed. Care should be taken not to encroach on the epiphyseal lines, if possible, especially in a young patient, since to damage them will inhibit any subsequent growth in the bones and lead to additional shortening. Another important point is to saw the bones at right angles to the shaft, or to cut the ends in such a way that when the bones are in contact there is a slight tendency to hyperextension. If care is not taken to do this flexion will subsequently occur at the point where the joint was, and the deformity, slight though it be at first, will gradually become more pronounced until the leg may become bent up almost at a right angle. This tendency to subsequent flexion is the commonest and most troublesome complication of excision of the knee. It has been suggested that it is due to the unopposed action of the biceps muscle, and this seems a reasonable hypothesis. Some surgeons say, therefore, that it is well to cut the tendon of this muscle at the time of the original operation. This small manœuvre is easily performed. Others advocate uniting the ends of the bone at the time of operation by silver wire: there is less to be said for this, since it is doubtful if the wire is able to prevent the flexion, and in the case of a tuberculous bone it is not advisable to drill it and put in a foreign body, which might quite possibly form the starting-point of a new focus of disease.

After the operation the limb should be put up on a back splint and two side splints, and treated in the same way as a compound fracture, which, indeed it is. Non-union sometimes follows, especially in adults, and a flail-like joint may result. This renders the limb so useless that it may become necessary to amputate it.

So far it has been assumed that the operation is being done for tuberculosis, but the writer has recently had occasion to do it for poliomyelitis. The patient was a boy aged seven who had an acute attack of poliomyelitis in infancy. The right leg only was affected, and the muscles which were picked out were those that acted upon the knee. These were completely useless, but movements at the hip and ankle were normal. There was no talipes. The limb in this state was quite useless to him, and excision of the knee was performed in order to give him a rigid column of support.

---

**Heart-failure in Stout Patients.**

One cannot expect to get good results in these cases unless the diet is cut down to a minimum. It may be difficult to induce a patient to submit at first, but after a week he will appreciate the relief, and the circulation will begin to respond to the medicinal treatment.—*Dr. Harry Campbell.*

**Paraldehyde.**

In spite of the invention of numerous new preparations, paraldehyde still remains one of the most efficient and safest of hypnotics. Its taste is perhaps its greatest drawback, but this may be disguised by infusum senegæ or syrup of orange. In the treatment of asthma the antispasmodic drugs are much more likely to be successful if the patient is able to get sufficient sleep, and in these cases paraldehyde is the best hypnotic to employ.

**After-treatment of Pleurisy.**

This simple exercise may be practised with advantage in order to promote the expansion of the lung which has been compressed by effusion. Let the patient sit on a chair and grasp with the hand of the sound side the rung under the seat of the chair, then let him breathe slowly and as deeply as possible. The cramped position of the sound side prevents this from taking too great a share in respiration, and tends to encourage the expansion of the opposite lung.

**High Blood-pressure.**

In middle age a pulse tension over 140mm. requires careful watching. Patients with high tension often possess a great capacity for work and almost untiring energy. Should the B.P. be high the amount of tea, coffee, and alcohol must be limited and protein food cut down. Any tendency to constipation must be corrected at once, preferably by blue pill and saline. An excellent measure is to give every morning in hot water the following powder: Potass. nitrate, grs. 10; potass. bicarb., grs. 10; sodium nitrite, gr. 1.

